# Iron elevates mesenchymal and metastatic biomarkers in HepG2 cells

**DOI:** 10.1038/s41598-020-78348-5

**Published:** 2020-12-14

**Authors:** Kosha J. Mehta, Paul A. Sharp

**Affiliations:** 1 Centre for Education, Faculty of Life Sciences and Medicine, King’s College London, London, UK; 2 Department of Nutritional Sciences, School of Life Course Sciences, Faculty of Life Sciences and Medicine, King’s College London, London, UK

**Keywords:** Biochemistry, Cell biology, Molecular biology

## Abstract

Liver iron excess is observed in several chronic liver diseases and is associated with the development of hepatocellular carcinoma (HCC). However, apart from oxidative stress, other cellular mechanisms by which excess iron may mediate/increase HCC predisposition/progression are not known. HCC pathology involves epithelial to mesenchymal transition (EMT), the basis of cancer phenotype acquisition. Here, the effect of excess iron (holo-transferrin 0–2 g/L for 24 and 48 h) on EMT biomarkers in the liver-derived HepG2 cells was investigated. Holo-transferrin substantially increased intracellular iron. Unexpectedly, mRNA and protein expression of the epithelial marker E-cadherin either remained unaltered or increased. The mRNA and protein levels of metastasis marker N-cadherin and mesenchymal marker vimentin increased significantly. While the mRNA expression of EMT transcription factors *SNAI1* and *SNAI2* increased and decreased, respectively after 24 h, both factors increased after 48 h. The mRNA expression of TGF-β (EMT-inducer) showed no significant alterations. In conclusion, data showed direct link between iron and EMT. Iron elevated mesenchymal and metastatic biomarkers in HepG2 cells without concomitant decrement in the epithelial marker E-cadherin and altered the expression of the key EMT-mediating transcription factors. Such studies can help identify molecular targets to devise iron-related adjunctive therapies to ameliorate HCC pathophysiology.

## Introduction

Independent of the underlying aetiology, untreated hepato-pathology in chronic liver diseases (CLDs) can progress through the overlapping stages of hepatitis, fibrosis and the frequently irreversible cirrhosis, often causing predisposition to hepatocellular carcinoma (HCC). HCC is the 5^th^ most common cancer in the world and the third highest cause of cancer-related deaths^1^, where transplantation and resection remain the only curative options. Mortality due to HCC is continuously increasing in Europe, U.S and Africa and is the ‘fastest rising cause of cancer-related deaths’ in the U.S^2^ with about 850,000 new cases every year worldwide^3^. Since HCC can emerge from various liver aetiologies, it is of a wide clinical benefit to understand the molecular mechanisms that lead to HCC predisposition and/or mediate its pathological acceleration.

Excess iron is commonly observed in several CLDs such as hereditary hemochromatosis, which manifests as severe systemic and tissue iron overload^4^. Here, the excess-iron-induced toxicity and inflammation causes a 200–fold increased risk for HCC^5^. Similarly, mild-moderate iron-excess is common in other CLDs like alcoholic liver disease, non-alcoholic fatty liver disease, non-alcoholic steatohepatitis, viral hepatitis and type 2 diabetes^6–10^. In these conditions, the exact role of iron (whether excess iron acts as a marker or mediator or both) in the pathophysiology remains an enigma.

The contribution of excess-iron-induced oxidative stress in liver pathologies has been studied extensively. However, excess iron may additionally activate other cellular mechanisms that may exacerbate the pathology. For example, excess iron activates the TGF-β pathway and enhances fibrogenesis, which may contribute to the development and progression of liver fibrosis/cirrhosis^11,12^. Such responses and mechanisms by which excess iron may initiate, mediate or accelerate disease progression to HCC need to be investigated. This can help identify molecular targets to develop adjunctive iron-related therapeutics and ameliorate HCC pathophysiology.

A mechanism by which cells can acquire cancerous and metastatic phenotype is epithelial to mesenchymal transition (EMT). While type 1 EMT is involved in organ development during embryogenesis and is an essential process, dysregulation in types 2 and 3 can have pathological implications^13,14^. In the latter, cells lose their epithelial characteristics marked by frequent downregulation of the epithelial marker E-cadherin and upregulate metastatic and mesenchymal markers N-cadherin and vimentin, respectively. This is accompanied by elevation in EMT-mediating transcription factors such as SNAIL and SLUG^13,14^. While the role of some iron-related proteins (e.g. DMT1, ferritin, ferroportin, STEAP3 and NDRG1) in modulating EMT has been studied^15^, whether excess iron per se directly induces or promotes EMT remains unknown.

Therefore, in this study, we examined the effect of iron on specific EMT features under the hypothesis that excess iron will reduce the levels of epithelial marker, elevate mesenchymal and metastatic markers and alter the gene expression of EMT-mediating transcription factors.

Accordingly, HepG2 cells were treated with increasing concentrations of holo-transferrin (holo-Tf) (0, 0.25, 0.5, 1 and 2 g/L) for 24 and 48 h (h). Cellular iron levels and several EMT-related biomarkers at gene and protein levels were examined; namely, gene expressions of *CDH1* (encoding the epithelial marker protein E-cadherin), *CDH2* (encoding metastasis marker protein N-cadherin), *VIM* (encoding mesenchymal protein vimentin), *SNAI1* and *SNAI2* (encoding EMT-mediating transcription factors SNAIL and SLUG) and *TGF-β* (EMT inducer), along with protein expressions of E-cadherin, N-cadherin and vimentin.

## Results

### Elevated intracellular iron in HepG2 cells

**Figure 1:**
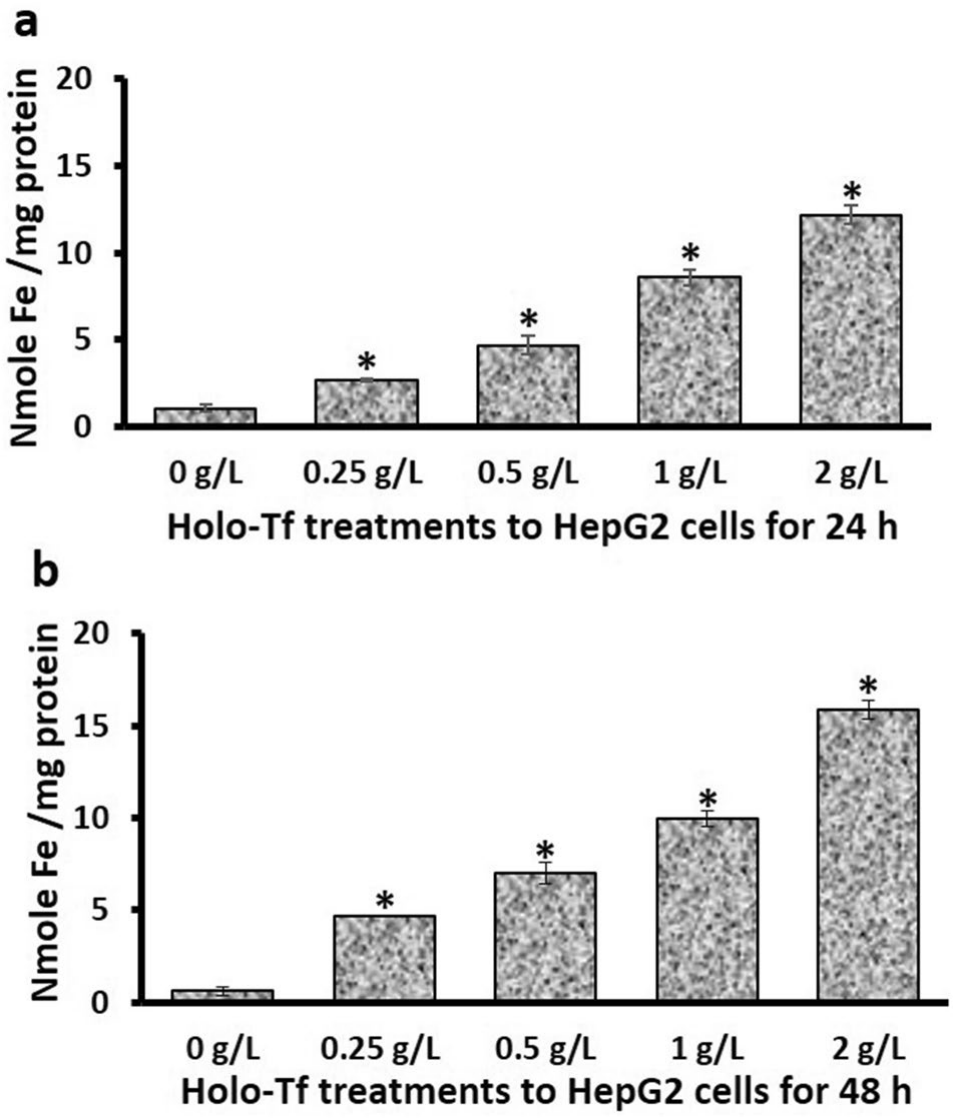
Holo-transferrin elevated cellular iron levels in HepG2 cells. HepG2 cells were treated with holo-Tf for 24 h (**a**) and 48 h (**b**), and cellular iron levels were determined by ICPMS. Data is presented as mean ± SEM (n = 3). *p <  = 0.01 compared to untreated control (0 g/L).

Firstly, we confirmed holo-Tf-induced intracellular iron excess in these cells (Fig. 1) to ensure that the observed effects on the epithelial and mesenchymal genes and proteins under study were exclusively due to iron treatments (in the form of holo-Tf). Data revealed that after 24 h, cellular iron levels in HepG2 cells increased by 2.7-, 4.7-. 8.6- and 12.2-fold with 0.25, 0.5, 1 and 2 g/L holo-Tf, respectively, compared to the untreated cells (0 g/L) (*p* < 0.01) (Fig. 1a). Higher increments were observed after 48 h where 0.25, 0.5, 1 and 2 g/L holo-Tf increased cellular iron levels by 7.8-, 11.6-, 16.6- and 26.5-fold, respectively (*p* < 0.01) (Fig. 1b). No alterations in cell morphology were observed during the entire duration of holo-Tf-treatments and for all concentrations.

### Limited iron-induced elevation of epithelial marker E-cadherin

**Figure 2:**
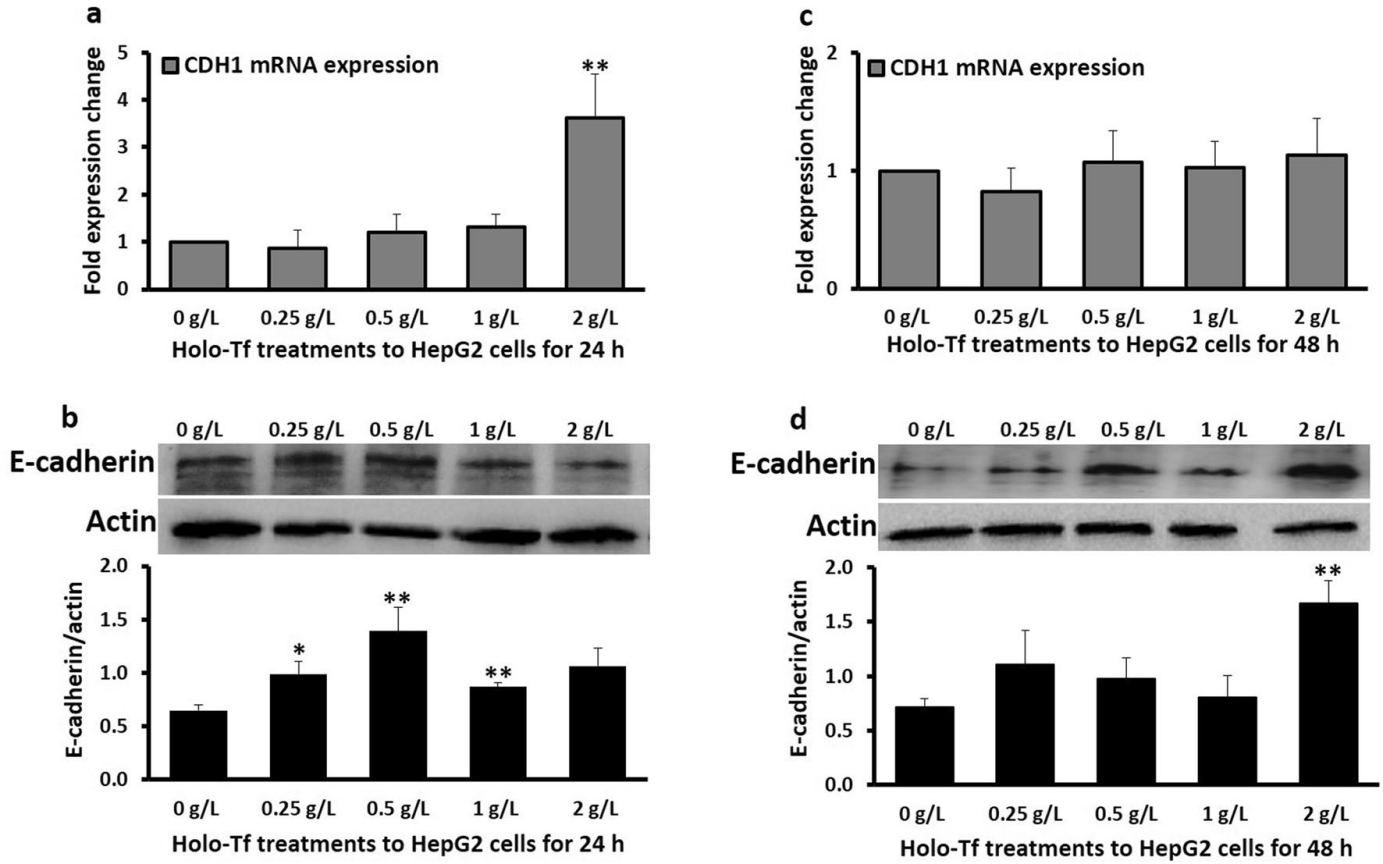
Effect of holo-transferrin on epithelial marker E-cadherin. HepG2 cells were treated with holo-Tf for 24 h (**a**, **b**) and 48 h (**c**, **d**). Gene expression of E-cadherin *(CDH1)* (**a**, **c**) and levels of E-cadherin protein (**b**, **d**) were determined by real-time PCR and western blot, respectively. Images are cropped versions. Full length images are in Supplementary Fig. 1. Protein intensity was analysed by the Image-J software available at the National Institute of Health (USA), downloadable from https://imagej.nih.gov/ij/download.html as of August 2020. Data is presented as mean ± SEM (n = 3–4). *p < 0.05 and **p <  = 0.03 compared to untreated control (0 g/L).

After 24 h, E-cadherin mRNA (*CDH1*) expression increased by 3.6-fold (*p* < 0.03) upon treatment with only 2 g/L holo-Tf, while other holo-Tf concentrations showed no significant effect (Fig. 2a). E-cadherin protein expression increased with 0.25 g/L (*p* = 0.05), 0.5 g/L (*p* = 0.03) and 1 g/L (*p* < 0.03) holo-Tf treatments (Fig. 2b). However, after 48 h, no significant alterations in *CDH1* mRNA expression were observed (Fig. 2c), and E-cadherin protein expression increased only upon 2 g/L holo-Tf treatment (*p* < 0.03) (Fig. 2d).

### Iron upregulated the metastatic mediator N-cadherin

**Figure 3:**
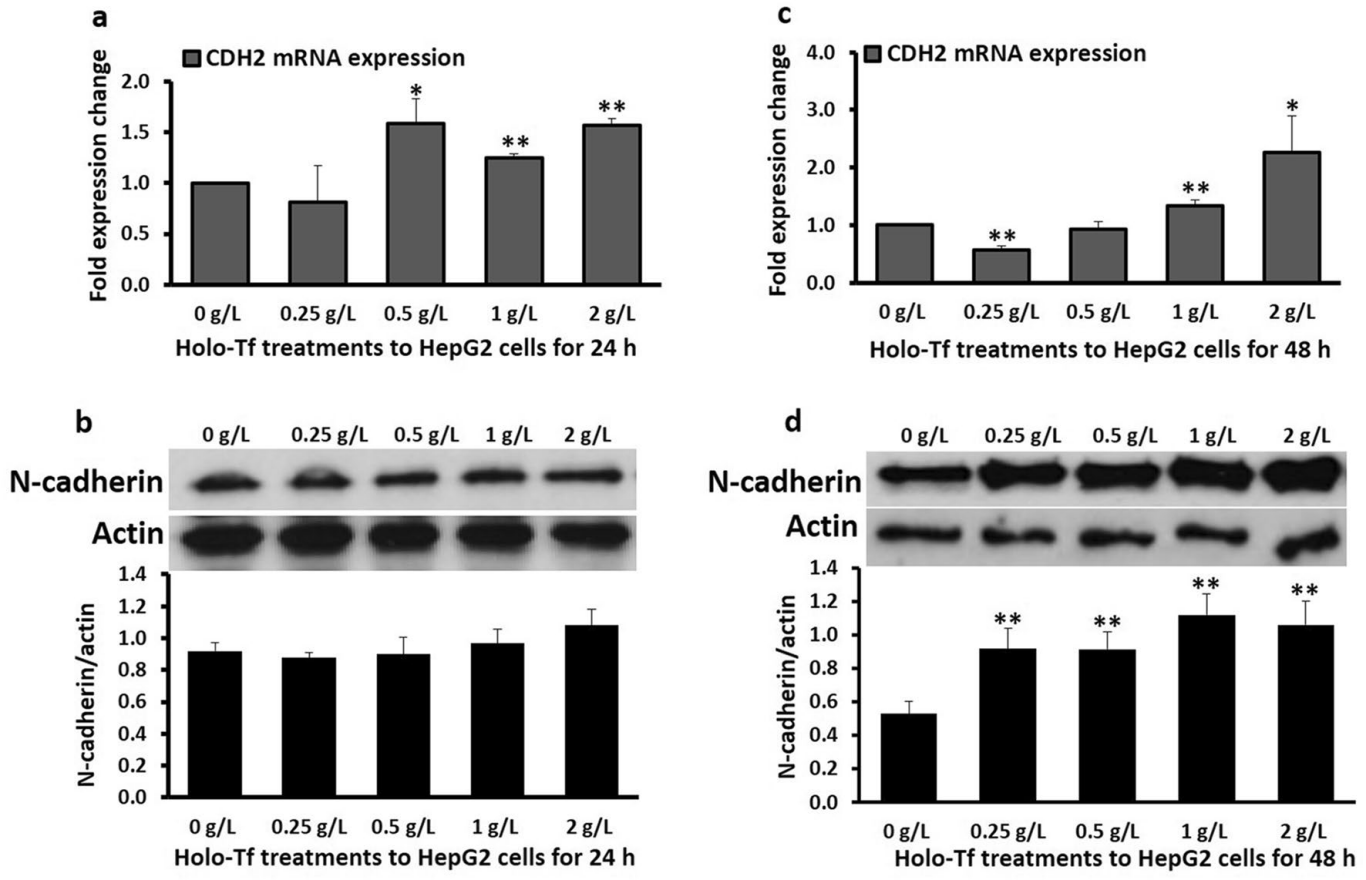
Effect of holo-transferrin on metastatic marker N-cadherin. HepG2 cells were treated with holo-Tf for 24 h (**a**, **b**) and 48 h (**c**, **d**). Gene expression of N-cadherin *(CDH2)* (**a**, **c**) and levels of N-cadherin protein (**b**, **d**) were determined by real-time PCR and western blot, respectively. Images are cropped versions. Full length images are in Supplementary Fig. 2. Protein intensity was analysed by the Image-J software available at the National Institute of Health (USA), downloadable from https://imagej.nih.gov/ij/download.html as of August 2020. Data is presented as mean ± SEM (n = 3–4). *p <  = 0.05 and **p <  = 0.03 compared to untreated control (0 g/L).

Holo-Tf treatments for 24 h increased N-cadherin mRNA *(CDH2)* expression. Increments were observed with 0.5 g/L (1.6-fold, *p* < 0.05), 1 g/L (1.2-fold, *p* = 0.03) and 2 g/L holo-Tf (1.6-fold, *p* < 0.03) (Fig. 3a). No significant alterations were observed in protein levels after 24 h (Fig. 3b). However, after 48 h, increments were observed in both, mRNA and protein levels. The mRNA expression increased upon 1 g/L and 2 g/L treatments (1.3-fold; *p* < 0.03 and 2.3-fold; *p* < 0.05, respectively) (Fig. 3c), while N-cadherin protein expression majorly increased upon treatments with all holo-Tf concentrations (*p* < 0.03) (Fig. 3d).

### Iron induced the expression of mesenchymal marker vimentin

**Figure 4:**
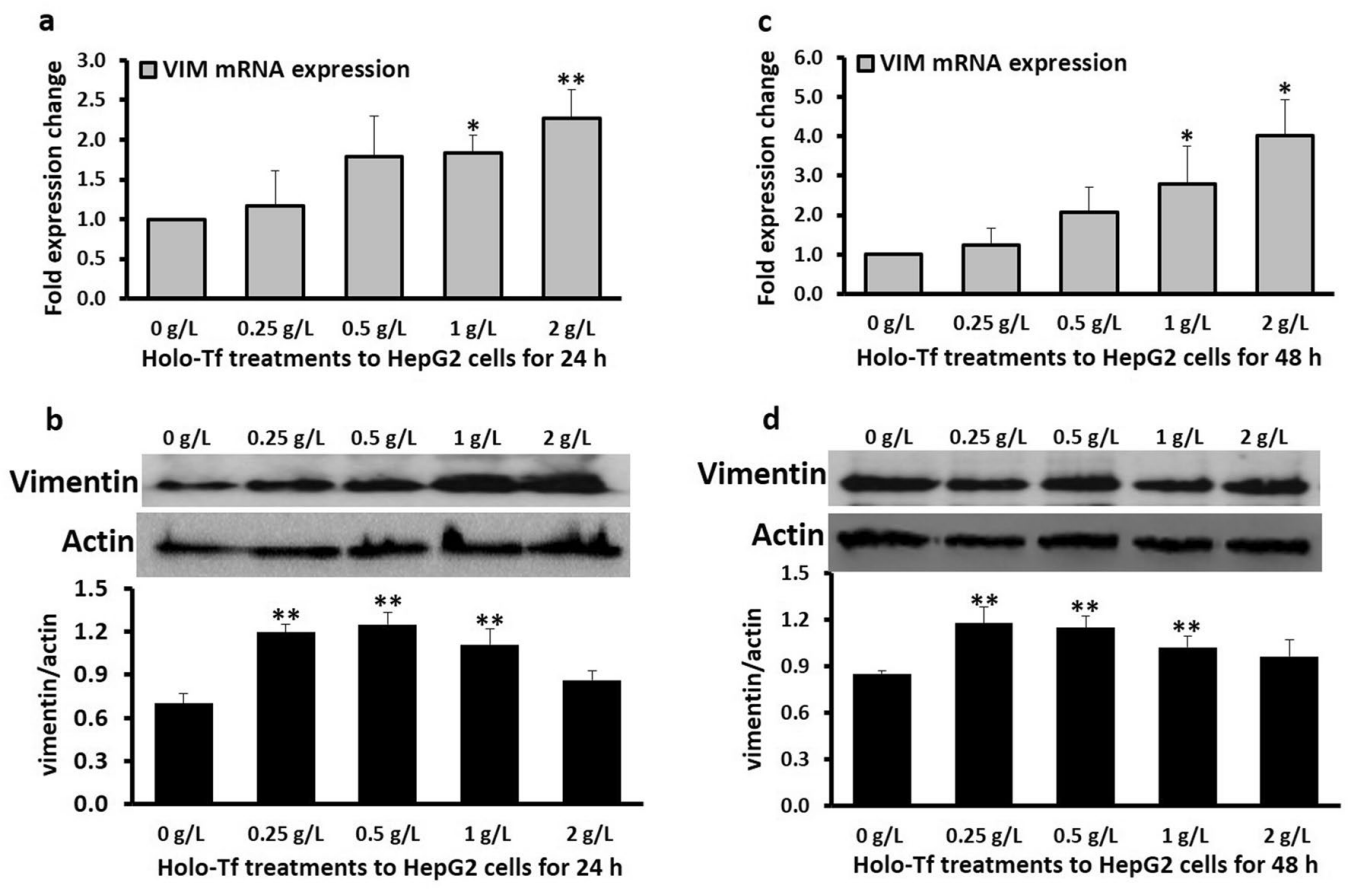
Effect of holo-transferrin on mesenchymal marker vimentin. HepG2 cells were treated with holo-Tf for 24 h (**a**, **b**) and 48 h (**c**, **d**). Gene expression of vimentin *(VIM)* (**a**, **c**) and levels of vimentin protein (**b**, **d**) were determined by real-time PCR and western blot, respectively. Images are cropped versions. Full length images are in Supplementary Fig. 3. Protein intensity was analysed by the Image-J software *pp* available at the National Institute of Health (USA), downloadable from https://imagej.nih.gov/ij/download.html as of August 2020. Data is presented as mean ± SEM (n = 3–4). * <  = 0.05 and **<  = 0.03 compared to untreated control (0 g/L).

Like N-cadherin mRNA *(CDH2)* expression*,* vimentin mRNA expression increased upon 1 g/L and 2 g/L treatments after 24 h (Fig. 4a) (1.8-fold; *p* < 0.05 and 2.3-fold; *p* < 0.03, respectively). Also, 0.25 g/L (*p* = 0.03), 0.5 g/L (*p* < 0.03) and 1 g/L (*p* < 0.03) holo-Tf elevated vimentin protein expression (Fig. 4b). Similarly, after 48 h, 1 g/L and 2 g/L holo-Tf further increased vimentin mRNA expression by 2.8-fold (*p* < 0.05) and 4-fold (*p* < 0.05), respectively (Fig. 4c). Vimentin protein expression also increased following treatments with 0.25 g/L (*p* = 0.03), 0.5 g/L (*p* < 0.03) and 1 g/L (*p* < 0.03) holo-Tf (Fig. 4d).

### Iron generally elevated EMT transcription factors

**Figure 5:**
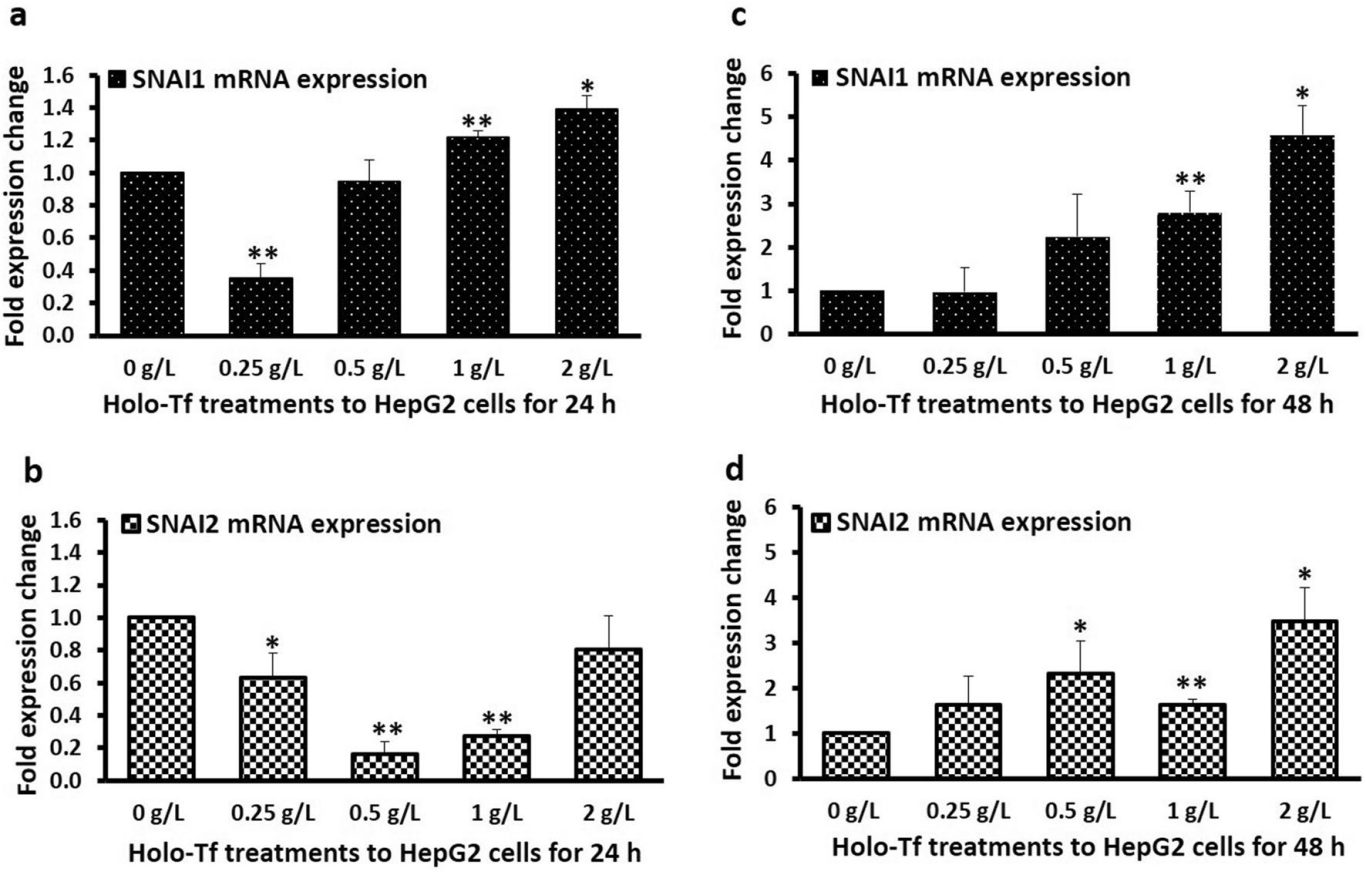
Effect of holo-transferrin on EMT transcription factors. The effect of holo-Tf treatments on the mRNA expression of EMT transcription factor SNAIL (*SNAI1*) (**a**, **c**) and SLUG (*SNAI2*) (**b**, **d**) for 24 h and 48 h have been shown. Data is presented as mean ± SEM (n = 3–4). *p <  = 0.05 and **p <  = 0.03 compared to untreated control (0 g/L).

After 24 h, holo-Tf treatments of 1 g/L and 2 g/L increased *SNAI1* expression (1.2-fold; *p* = 0.03 and 1.4-fold; *p* < 0.05, respectively) (Fig. 5a), while 0.5 and 1 g/L treatments decreased *SNAI2* expression (0.2-fold; *p* < 0.03 and 0.3-fold; *p* < 0.03, respectively) (Fig. 5b). Low concentration of holo-Tf (0.25 g/L) reduced the expressions of both, *SNAI1* and *SNAI2* (0.4-fold; *p* < 0.03 and 0.6-fold; *p* < 0.05, respectively) (Fig. 5a,b). Unlike this, after 48 h, 1 g/L and 2 g/L holo-Tf increased the expressions of both, *SNAI1* (2.8-fold; *p* < 0.03 and 4.6-fold; *p* < 0.05) and *SNAI2* (1.6-fold; *p* < 0.03 and 3.5-fold; *p* < 0.05), where *SNAI2* expression also increased upon 0.5 g/L holo-Tf treatment (*p* < 0.05) (Fig. 5c,d).

### Effect of iron treatment on TGF-β mRNA expression

**Figure 6:**
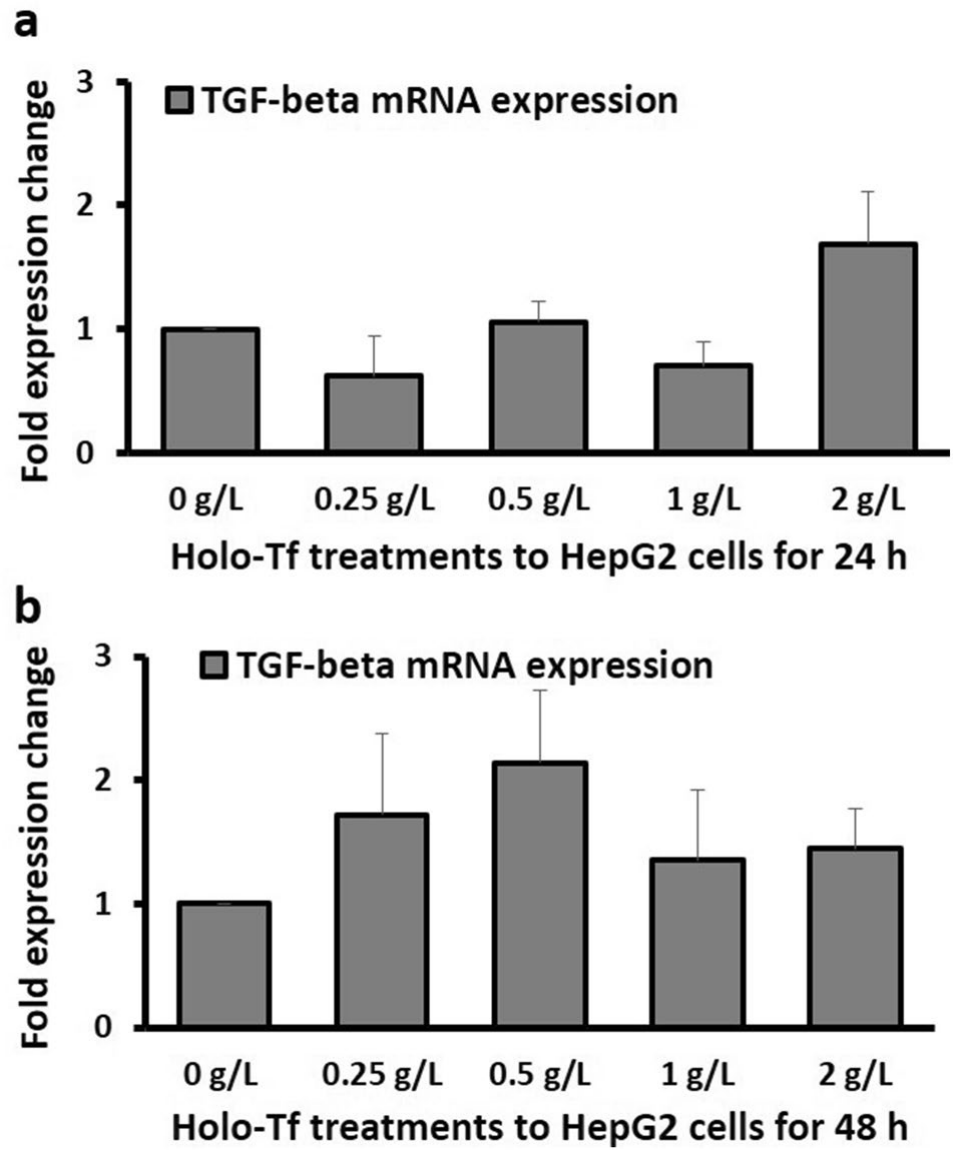
Effect of holo-transferrin on *TGF-β* expression. The effects of holo-Tf treatments on the mRNA expression of *TGF-β* for 24 h (**a**) and 48 h (**b**) have been shown. Data is presented as mean ± SEM (n = 3).

No significant alterations were observed in TGF-β mRNA expression following holo-Tf treatments (Fig. 6). Statistically insignificant increments in expression were observed upon treatment with 2 g/L for 24 h, and 0.25 g/L and 0.5 g/L treatments for 48 h (Fig. 6).

## Discussion

Liver iron loading is commonly observed in several CLDs where untreated pathology may progress to HCC^11^. HCC patients frequently demonstrate liver iron overload^16–18^ and its correlation with the risk for HCC development is well-established^19^. However, apart from the iron-induced free-radical damage, other mechanisms by which excess iron may increase this risk and cause HCC predisposition and/or accelerate its progression are unclear. EMT is a process that mediates the acquisition of a cancerous and metastatic phenotype^13^. Whether excess iron per se induces or enhances EMT and thereby contributes to cancer initiation, sustenance and progression remains enigmatic. Therefore, we investigated the effect of a range of iron (holo-Tf) concentrations (0, 0.25, 0.5, 1 and 2 g/L) for 24 and 48 h on core EMT biomarkers in HepG2 cells.

Cellular iron-loading in HepG2 cells was confirmed (Fig. 1), which showed no noticeable alterations in cell morphology during the entire course of this study. This is similar to previous studies where treatment of HepG2 cells with higher concentrations of holo-Tf did not noticeably change cell morphology^20,21^.

Then, we assessed the core biomarkers related to EMT. E-cadherin is an epithelial cell-adhesion molecule and its down regulation is a characteristic of EMT and metastasis^14^. Reduced tissue expression of E-cadherin has been described in HCC^22,23^ and impaired E-cadherin expression has been shown to promote liver carcinogenesis in men and mice^24^. Accordingly, we hypothesised that holo-Tf treatments would decrease E-cadherin mRNA and protein expression. However, here, the mRNA expression generally remained unaltered and also showed some increments in protein expression (Fig. 2). The elevations in E-cadherin expression are not completely surprising because this has been observed previously in some clinical samples (liver tissues from HCC patients), where E-cadherin expression was markedly higher than control non-tumorous liver samples^25^. Such responses could be because of the paradoxical role of E-cadherin that suggest its involvement in some tumoral steps^25^. Generally, it is recognised that carcinoma involves uncontrolled growth of epithelial cells accompanied by reduction in cell–cell adhesion and increased invasiveness, but E-cadherin expression has shown a positive correlation with the invasive capacity of tumour cells^25^ and is deemed necessary for HCC cell lines to mediate intrahepatic metastasis^26^. Thus, the observed increments in E-cadherin expression (Fig. 2) indicate the contribution of excess iron in the fore-steps towards a metastatic phenotype.

Upon treatments with some holo-Tf concentrations, E-cadherin mRNA and protein expression remained unaltered (Fig. 2). This is in line with previous clinical studies where E-cadherin expression in HCC patients’ tumour samples without cirrhosis and in hepatocellular adenoma was similar to that in normal liver samples^27^, thus reiterating the diversity in E-cadherin responses based on the stages and pathological severity of HCC. Lack of alteration in E-cadherin expression could be partly because cadherin switching (downregulation of E-cadherin along with concomitant upregulation of N-cadherin) includes scenarios where N-cadherin expression can increase without significant changes in E-cadherin expression^28^. Therefore, it is possible that excess iron may increase mesenchymal features without mandatory decrements in E-cadherin expression, as observed here (Figs. 2, 3, 4).

For both E-cadherin and N-cadherin, the pattern of mRNA expression at 24 h matched the pattern of their protein expression at 48 h (Fig. 2a,d, and Fig. 3a,d), suggesting a time-delay between their mRNA and protein expressions. While such a time-delay/gap between the mRNA and protein expression is not surprising and is determined by several factors including the size of the protein, post-translation modifications etc., elevation in E-cadherin protein at 24 h followed by a general lack of elevation in E-cadherin mRNA and protein expression at 48 h (Fig. 2c,d) suggests a possibility that prolonged holo-Tf treatment beyond 48 h may either have no effect or decrease E-cadherin expression, as hypothesised. The increment in E-cadherin mRNA expression upon 2 g/L treatment (Fig. 2a) and elevations in protein expression at the 24-h time-point (Fig. 2b) could be temporary and a counteractive protective mechanism to tackle the increasing extracellular and intracellular holo-Tf concentrations (Fig. 1) and prevent the downregulation of this epithelial marker under excess iron conditions to maintain cell–cell connections in the early stages of iron loading.

Contrasting E-cadherin, N-cadherin is primarily expressed in mesenchymal and cancer cells^14^, and a switch from E-cadherin to N-cadherin is a characteristic of invasion and metastasis^28^. The iron-induced upregulation of N-cadherin at mRNA and protein levels (Fig. 3) (as hypothesised) and E-cadherin responses (Fig. 2) with the contextual diversity of its roles together suggests that excess iron may execute a metastatic role by modulating these biomarkers and promote or help maintain invasiveness in HCC. Furthermore, since mesenchymal features are typically upregulated during EMT^13^, here, we hypothesised that holo-Tf treatments would upregulate the expression of the mesenchymal marker vimentin. As expected, data showed holo-Tf-induced increments in vimentin expression at mRNA and protein levels (Fig. 4). Vimentin overexpression plays an important role in HCC metastasis^29^ and the data here clearly demonstrates excess-iron-induced enhancement in this mesenchymal marker, which could contribute to metastasis in HCC. Such iron-induced increment in vimentin expression has been observed previously in hepatic stellate cells^12^, which supports the role of excess iron in promulgating a mesenchymal phenotype, aggravating liver fibrosis and increasing HCC predisposition.

Further on, to understand the mechanistic aspects of the observed holo-Tf induced-alterations, we examined the mRNA expression of EMT-mediating transcription factors *SNAI1* and *SNAI2* (encoding proteins SNAIL and SLUG, respectively). These are the key mediators of TGF-β-induced EMT^30^ that can downregulate E-cadherin expression^31–33^, while upregulating the expressions of N-cadherin^34^ and vimentin^35^. Here, holo-Tf treatments generally upregulated these transcription factors (Fig. 5a,c,d) demonstrating their role in iron-induced EMT. While the cause of downregulation of *SNAI2* expression at 24 h (Fig. 5b) needs to be investigated, it must be noted that the binding affinities of these transcription factors to E-cadherin (*CDH1)* promoter (and thereby their abilities to alter gene expression) are different, which cause subtle differences and non-equivalence between their roles^36^. This may have contributed to the different response of *SNAI2* expression at 24 h (Fig. 5b), while all other data showed a general iron-induced elevation (Fig. 5a,c,d). Probably, this major iron-induced downregulation of *SNAI2* at 24 h (Fig. 5b) nullified the effect of subtle upregulations of *SNAI1* (Fig. 5a) and contributed to the iron-induced upregulation of E-cadherin at this time point (Fig. 2a,b). Simultaneously, it may have contributed to the lack of holo-Tf-induced upregulation of N-cadherin protein at the 24 h time-point (Fig. 3b). However, after 48 h, the upregulation of these transcriptional factors (Fig. 5c,d) coincided with the upregulations of both N-cadherin (Fig. 3c,d) and vimentin (Fig. 4c,d). This suggests that like in case of TGF-β-induced EMT, iron-induced alterations in the epithelial and mesenchymal markers may be partly mediated via these factors.

Thus, it is possible that EMT, which mediates the acquisition of a cancerous phenotype and pathological progression of HCC, may be fully or partly facilitated and accelerated by excess iron in HCC patients; particularly in cases where the HCC emerged from hereditary hemochromatosis. Further studies are essential to confirm this. Contextually, although EMT in liver fibrosis may be controversial, excess-iron-accelerated EMT is possible and could have substantial pathological implications in HCC.

In this study, although iron enhanced specific EMT characteristics, TGF-β expression did not alter significantly (Fig. 6). Therefore, the role of excess iron in inducing TGF-β expression in HepG2 cells could not be clarified. As it is, TGF-β exhibits a dual and context-dependent role in cancer, whereby it is protective in the initial stages, but promotes tumour cell invasion and metastasis in the later stages^37^. This makes it challenging to delineate its exact role in EMT under excess iron conditions.

Since HCC can emerge from different aetiologies and is a growing concern world-wide, understanding its molecular mechanisms is crucial. This can help identify molecular targets and formulate better strategies to evade HCC predisposition and decelerate disease progression. For example, the EMT markers N-cadherin^38^ and vimentin^39,40^ have been recognised as potential targets for cancer therapy. Data here shows the involvement of excess iron in upregulating these markers. Therefore, such investigations can help formulate iron-related adjunctive therapeutic strategies that may prevent these iron-induced alterations and further inform oncotherapy via chelators and iron oxide nanoparticles^41^.

Hepcidin is the master regulator of iron homoeostasis^42^. However, we did not measure it on this occasion because hepcidin expression as a standalone or the effect of iron on hepcidin in HepG2 cells has been previously examined by various groups^20,21,43–51^. In addition, we already know that hepcidin expression is diminished in hepatocellular carcinoma, unlike other cancers, and its reduction contributes to the aggressiveness of the disease^52,53^. We also know the significance of hepcidin-ferroportin axis in metastasis where hepcidin is thought to play a contributory role in promoting cancer cell homing and fostering metastasis^54^. Thus, re-examining the effect of iron on hepcidin expression in HepG2 cells would neither be novel nor contribute substantially to our understanding of the effect of iron on EMT biomarkers, which was the aim of this study.

Here, we focussed on the EMT signature because although the effect of excess-iron-induced oxidative stress via acceleration of the Fenton reaction in well-understood, other mechanisms by which iron may increase the predisposition for hepatocellular carcinoma and/or accelerate its progression are unclear. In another perspective, despite the prevalence of excess hepatic iron in several chronic liver diseases^11^, whether iron promotes EMT in the liver has not been investigated yet. Here, we hypothesised that iron may activate EMT mechanisms and thereby contribute to carcinogenesis. Our data shows that iron does have an effect on EMT biomarkers, which is novel in the context of liver.

Holo-Tf was chosen for iron treatments because it is a physiological relevant form of iron. Transferrin-bound iron circulates in the body and supplies iron to all cells by binding to transferrin receptors on cell surfaces. This is a well-studied, regulated mechanism of cellular iron uptake^55,56^, unlike the uptake of non-transferrin bound iron, which is usually achieved *invitro* via treatment of cells with cheaper iron sources like ferrous sulphate or ferric ammonium citrate. Another aspect in choosing holo-Tf as the iron source was the significance of iron saturation of transferrin in the body. Transferrin saturation can be high in several conditions like hereditary hemochromatosis^57^ where the iron overload can dramatically increase the risk for hepatocellular carcinoma^58^, in alcoholic and non-alcoholic liver disease^59^ where iron elevation is believed to contribute to disease pathology, and even in general adult population, where it can increase the risk for all-cause mortality^60^. Thus, choosing holo-Tf as the iron treatment source was to maintain physiological relevance, and choosing to treat the cells with not one or two, but a range of holo-Tf concentrations was an attempt to reflect and cover the varying degrees of iron saturation of transferrin under physiological and pathological conditions. Thus, we chose holo-Tf as the iron source.

In future, examining protein levels of TGF-β, SNAI1 (SNAIL) and SNAI2 (SLUG), and assessing the transcriptional activities of the transcription factors would be useful in understanding the growing picture of the effect of iron on EMT. Additionally, it would be interesting to determine whether the usage of iron chelators like deferoxamine reversed the holo-Tf-induced EMT effects; similar to the reversal of holo-Tf-induced fibrogenic effects by deferoxamine in murine hepatic stellate cells^12^. This would provide direct translational applications for iron-overload conditions. This preliminary study could be extended by examining the effect of iron on EMT in an additional cancer cell line to confirm that these observations were not limited to HepG2 cells. Replicating these experiments in an animal model of iron-overload would be the subsequent step.

## Conclusion

Apart from oxidative stress, other cellular mechanisms by which excess iron could promote cancer development and progression are not fully deciphered. To our knowledge, for the first time we provide first proof of concept that excess iron in the form of holo-Tf enhances mesenchymal and metastatic characteristics (namely, N-cadherin and vimentin) in HepG2 cells, accompanied by lack of E-cadherin downregulation. These responses may be partially mediated via iron-induced alterations in the expressions of EMT transcription factors (*SNAI1* and *SNAI2*). Thus, this data shows a direct link between iron and EMT and indicates that excess-iron-induced cancerous phenotype could be acquired by cellular mechanisms other than oxidative stress. Knowledge of such mediators and mechanisms is clinically significant as it may identify molecular targets and help in formulating iron-related therapies that could reduce HCC predisposition in several liver pathologies.

## Methods

### Cell culture and treatments

HepG2 cells were maintained in Eagle’s minimal essential medium (EMEM)

(Sigma-Aldrich, UK) with 10% foetal bovine serum (FBS) (Sigma-Aldrich, UK), 1% penicillin–streptomycin (Sigma-Aldrich, UK), 1% glutamine (Gibco, UK) and 1% non-essential amino acids (Sigma-Aldrich, UK)^20,48^. Trypsinization was performed with 0.25% Trypsin–EDTA (Gibco, UK). 500 × 10^3^ cells were seeded per well in 6-well plates and incubated in maintenance medium for 48 h to allow cell-multiplication and cell-acclimatisation. Then, the cells were maintained in 2% FCS for 24 h. Following this, the cells were treated with 0, 0.25, 0.5, 1 and 2 g/L holo-Tf (BBI solutions, UK). Various parameters were assessed after 24 h and 48 h.

### Determination of cellular iron content

Cellular iron content was determined by inductively coupled plasma mass spectrometry (ICP-MS), as published recently^61^.

### Gene expression analysis

Gene expression was examined as described in previous studies^12,21^. Essentially, cells were washed with phosphate buffered saline (PBS), treated with TRI reagent (Sigma Aldrich, UK) and the RNA was extracted as per manufacturer’s instructions. Reverse transcription and cDNA synthesis of 1000 ng RNA was conducted using High Capacity CDNA reverse transcription kit (Applied Biosystems, UK), as recommended by the manufacturer. Gene expression was studied via real-time PCR using FAST SYBR green master mix (Applied Biosystems, UK) on Applied Biosystems 7500 instrument (Supplementary Table S1). Data were analysed by the relative quantification method and expressed as 2^-∆∆Ct^ indicating fold expression change^62^.

### Assessment of protein biomarkers

Proteins E-cadherin, N-cadherin and vimentin were assessed by western blotting^12^. Cells were washed with PBS and lysed with RIPA buffer (Sigma-Aldrich, UK) containing Complete-Mini Protease inhibitor cocktail (Roche, UK), as per manufacturer’s instructions, and as previously performed^12^. Cell extracts were centrifuged for 5 min at 13,000 rpm and the supernatant was electrophoresed on Tris–glycine gels. Following protein transfer to nitrocellulose membrane, membranes were probed with primary antibodies overnight in a cold room, followed by treatment with HRP-conjugated detection antibodies for 1 h at RT on shaker; as previously performed^12^ (Supplementary Table S2). The blots were probed with Clarity Western ECL substrate (Bio-Rad, USA) and the protein bands were observed on X-ray films. Protein intensity was analysed by the Image-J software available at the National Institute of Health (USA), downloadable from https://imagej.nih.gov/ij/download.html as of August 2020. Protein expression was normalised to beta actin levels.

### Statistical analysis

Data was analysed using student’s T-test (two-tailed distribution, two sample, and unequal variance). The level of significance was set at *p* < 0.05. Data was presented as mean ± SEM (n = 3–4).

## Supplementary information

Supplementary Information.

## Abbreviations

CLD: Chronic liver disease
EMT: Epithelial mesenchymal transition
HCC: Hepatocellular carcinoma
Holo-Tf: Holo-transferrin
ICP-MS: Inductively coupled plasma mass spectrometry
