# Supplementary material for: Iron elevates mesenchymal and metastatic biomarkers in HepG2 cells
Source: Sci Rep. 2020 Dec 14;10:21926. doi: 10.1038/s41598-020-78348-5 (PMC7736862; doi:10.1038/s41598-020-78348-5)
Supplement: Supplementary file 1 — Supplementary Information. [file 41598_2020_78348_MOESM1_ESM.pdf]

Supplementary file

Iron elevates mesenchymal and metastatic biomarkers in HepG2 cells

Kosha J. Mehta[1*] and Paul A. Sharp[2]
[1]Centre for Education, Faculty of Life Sciences & Medicine, King's College London, London, UK.
[2]Department of Nutritional Sciences, School of Life Course Sciences, Faculty of Life Sciences & Medicine, King's College London, London, UK.
*Corresponding author: Kosha.mehta@kcl.ac.uk

Supplementary Table S1: Primers used for gene expression analysis

Genes	Primers 5' to 3'
**CHD1**	TGAAGGTGACAGAGCCTCTGGAT and TGGGTGAATTCGGGCTTGTT (1)
**CDH2**	CACCGTGGTCAAACCAATCG and GAAGCCCTTCTTCTTGGCGA
**VIM**	AACGCCAGATGCGTGAAATG and AGAAATCCTGCTCTCCTCGC
**TGF-β1**	CTGGCGATACCTCAGCAACC and CGGTAGTGAACCCGTTGATGT
**SNAI1**	TAATCCAGAGTTTACCTTCCAGCA and TCCCACTGTCCTCATCTGACA
**SNAI2**	CGGACCCACACATTACCTTGTGTTT and CACAGCAGCCAGATTCCTCATGTTT (2)
**β-2 microglobulin**	CCACTGAAAAAGATGATGATGCCT and CCAATCCAAATGCGGCATCTTCA (3)

References

1. Lee H-J, Su Y, Lui W-Y, Chau G-Y, Yin P-H, Lee H-C, et al. Peroxisome proliferator-activated receptor gamma coactivator-1 alpha (PGC-1α) upregulated E-cadherin expression in HepG2 cells. FEBS Lett. 2008 Mar 5;582(5):627–34.

2. Satow R, Hirano T, Batori R, Nakamura T, Murayama Y, Fukami K. Phospholipase Cδ1 induces E-cadherin expression and suppresses malignancy in colorectal cancer cells. Proc Natl Acad Sci U S A. 2014 Sep 16;111(37):13505–10.

3. Sharp PA, Clarkson R, Hussain A, Weeks RJ, Morison IM. DNA methylation of hepatic iron sensing genes and the regulation of hepcidin expression. PLoS ONE [Internet]. 2018 May 17 [cited 2019 Jun 14];13(5). Available from: https://www.ncbi.nlm.nih.gov/pmc/articles/PMC5957407/

Supplementary Table S2: Antibodies used during Western blotting

Proteins	Primary antibody	Detection antibody
**E-cadherin**	Recombinant Anti-E Cadherin antibody (ab133597) (Abcam) (1:1000)	P0448 Goat Anti-Rabbit Immunoglobulins/HRP (affinity isolated) (1:10,000)
**N-cadherin**	Anti-N Cadherin antibody (ab18203) (Abcam) (1:1000)	P0448 Goat Anti-Rabbit Immunoglobulins/HRP (affinity isolated) (1:10,000)
**Vimentin**	Anti-Vimentin antibody - Cytoskeleton Marker (ab45939) (Abcam) (1.5:1000)	P0448 Goat Anti-Rabbit Immunoglobulins/HRP (affinity isolated) (1:3000)
**β-actin**	Anti-β-Actin antibody, Mouse monoclonal A1978 (Sigma - Aldrich) (1:10,000)	Goat Anti-Mouse IgG, H&L Chain Specific Peroxidase Conjugate (MERCK) (1:1000)

Supplementary Fig. 1

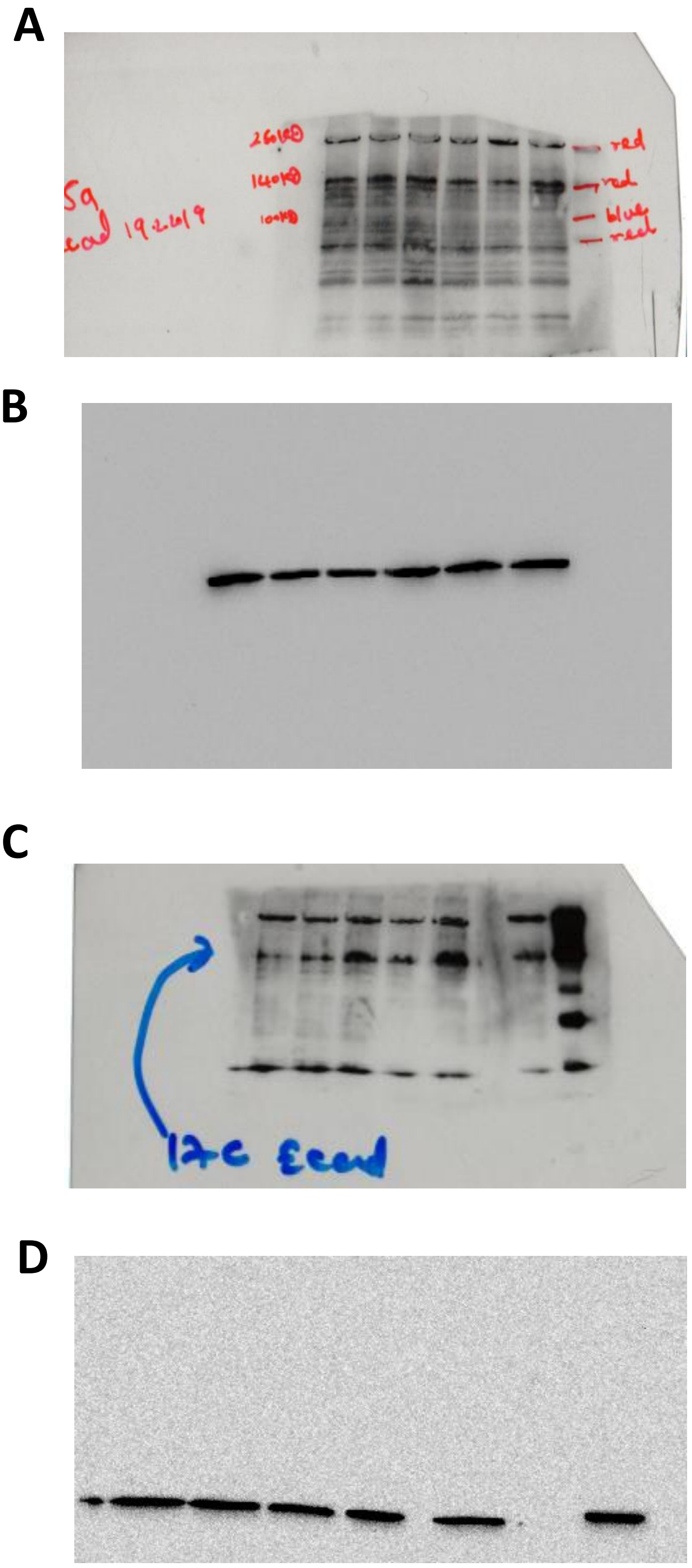

Supplementary Fig.1 Membranes probed with E-cadherin and actin antibodies

A: Image of membrane probed with E-cadherin antibodies, reflecting holo-Tf treatments for 24 h. B: Image of the same membrane probed with actin antibodies, reflecting holo-Tf treatments for 24 h. C: Image of membrane probed with E-cadherin antibodies, reflecting holo-Tf treatments for 48 h. D: Image of the same membrane probed with actin antibodies, reflecting holo-Tf treatments for 48 h.

Supplementary Fig. 2

A

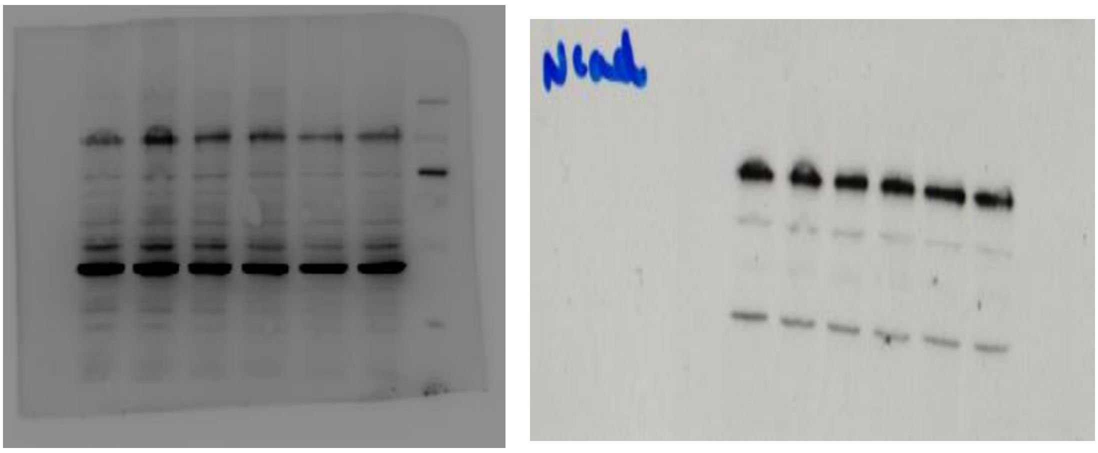

B

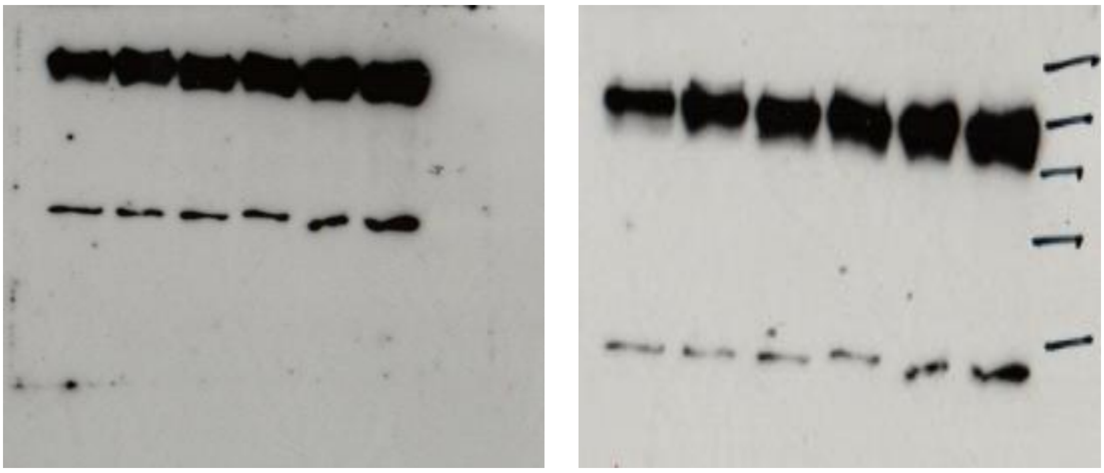

Supplementary Fig.2 Membranes probed with N-cadherin and actin antibodies

A: Image of the membrane probed with N-cadherin antibodies followed by probing of the same membrane with actin antibodies, reflecting holo-Tf treatments for 24 h. B: Image of the membrane probed with N-cadherin antibodies followed by probing of the same membrane with actin antibodies, reflecting holo-Tf treatments for 48 h.

Supplementary Fig. 3

A

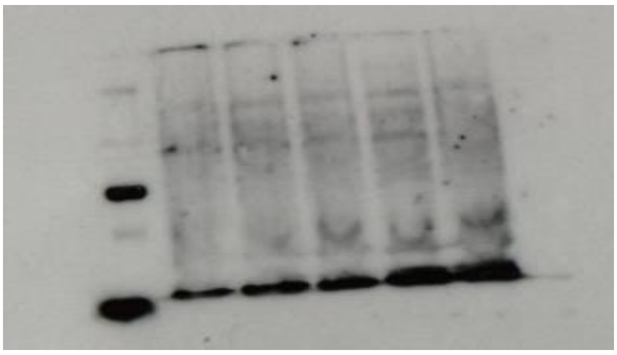

B

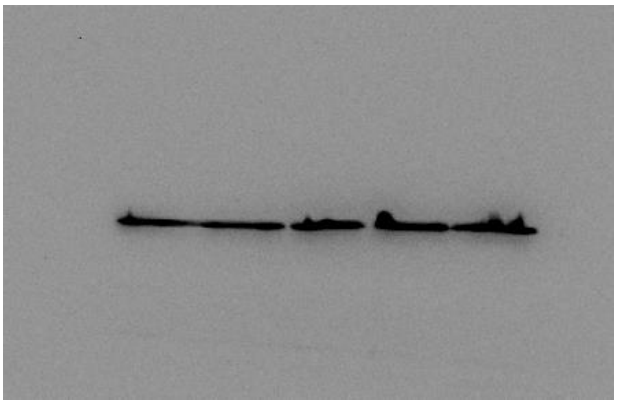

C

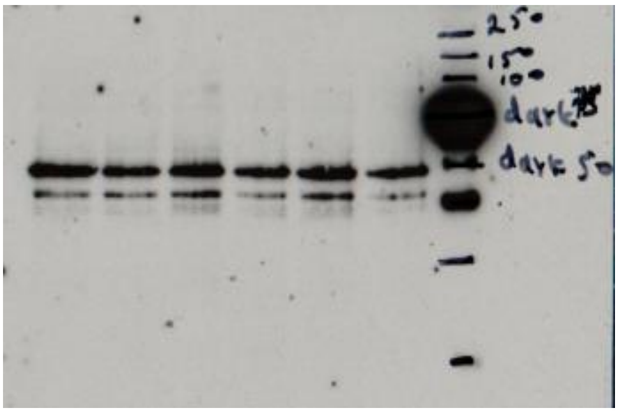

D

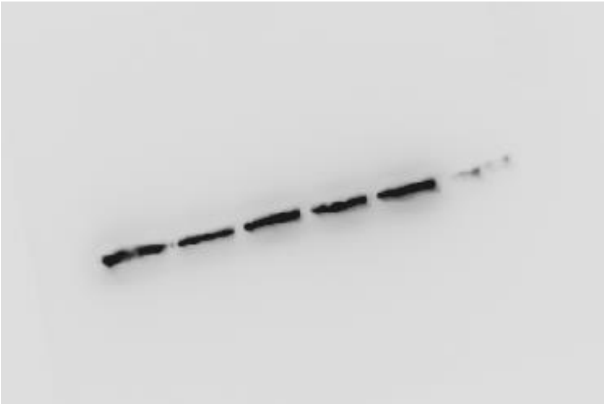

Supplementary Fig.3 Membranes probed with vimentin and actin antibodies

A: Image of membrane probed with vimentin antibodies, reflecting holo-Tf treatments for 24 h. B: Image of the same membrane probed with actin antibodies, reflecting holo-Tf treatments for 24 h. C: Image of membrane probed with vimentin antibodies, image reflecting holo-Tf treatments for 48 h. D: Image of the same membrane probed with actin antibodies, reflecting holo-Tf treatments for 48 h.
